# PGC1s and Beyond: Disentangling the Complex Regulation of Mitochondrial and Cellular Metabolism

**DOI:** 10.3390/ijms22136913

**Published:** 2021-06-27

**Authors:** Lara Coppi, Simona Ligorio, Nico Mitro, Donatella Caruso, Emma De Fabiani, Maurizio Crestani

**Affiliations:** Dipartimento di Scienze Farmacologiche e Biomolecolari, Università degli Studi di Milano, via Balzaretti 9, 20133 Milano, Italy; lara.coppi@unimi.it (L.C.); simona.ligorio@unimi.it (S.L.); nico.mitro@unimi.it (N.M.); donatella.caruso@unimi.it (D.C.)

**Keywords:** mitochondria, metabolic regulation, mitochondrial disorders

## Abstract

Metabolism is the central engine of living organisms as it provides energy and building blocks for many essential components of each cell, which are required for specific functions in different tissues. Mitochondria are the main site for energy production in living organisms and they also provide intermediate metabolites required for the synthesis of other biologically relevant molecules. Such cellular processes are finely tuned at different levels, including allosteric regulation, posttranslational modifications, and transcription of genes encoding key proteins in metabolic pathways. Peroxisome proliferator activated receptor γ coactivator 1 (PGC1) proteins are transcriptional coactivators involved in the regulation of many cellular processes, mostly ascribable to metabolic pathways. Here, we will discuss some aspects of the cellular processes regulated by PGC1s, bringing up some examples of their role in mitochondrial and cellular metabolism, and how metabolic regulation in mitochondria by members of the PGC1 family affects the immune system. We will analyze how PGC1 proteins are regulated at the transcriptional and posttranslational level and will also examine other regulators of mitochondrial metabolism and the related cellular functions, considering approaches to identify novel mitochondrial regulators and their role in physiology and disease. Finally, we will analyze possible therapeutical perspectives currently under assessment that are applicable to different disease states.

## 1. Introduction

Living organisms require energy and building blocks to elaborate and actuate specialized functions in each cell type. To this aim, metabolism represents the “core business” for different cell types as it provides metabolic intermediates and high energy compounds to establish the thermodynamic favorable conditions required for key biological processes to occur. In all cells, the exchange “bitcoin” to provide energy is most often ATP, a molecule containing phosphoanhydride bonds that, upon hydrolysis, can release sufficient energy to allow reactions. Mitochondria are the main site for ATP synthesis. They are key subcellular organelles present in all cells of the body apart from red blood cells in humans and animals, where many vital biochemical reactions take place. They use reducing metabolites and coenzymes, such as NADH and FADH_2_, which are derived from metabolic processes, to execute oxidative phosphorylation (OxPHOS) through the electron transport chain (ETC) and generate ATP to support various cellular functions. At the same time, reactive oxygen species (ROS), by-products of mitochondrial respiration, are produced and may damage various components in cells. Thus, mitochondria are essential for the maintenance of normal physiological functions [1]. Current knowledge on the number of proteins required for mitochondrial function stands at 1.158 [2]. Most of these proteins are encoded by nuclear DNA (nDNA), and only 13 are encoded by mitochondrial DNA (mtDNA). Thus, mitochondria are under the dual genetic control of both mitochondrial and nuclear genomes. Given their diverse functions, only ∼10% of total mitochondrial proteins are directly involved in OxPHOS and ATP production [3]. The remaining mitochondrial proteins are involved in the assembly of the complexes, the maintenance and expression of mtDNA, intraorganellar protein synthesis, and mitochondrial dynamics [4]. Nuclear-encoded mitochondrial proteins are translated in the cytoplasm and then imported into mitochondria through an intricate system [5].

All metabolic pathways, either mitochondrial, cytosolic or others, are interconnected and their fluxes are finely orchestrated depending on the needs of the organism in response to a wide variety of environmental cues that require an adaptation. Many differential strategies to fine tune metabolic pathways assure the proper response. They act at different levels, including the modulation of the levels of rate-limiting enzymes of metabolic pathways and of their activity via posttranslational modifications, interactions with other proteins or allosteric modulators. Altogether, these mechanisms contribute to maintain cellular homeostasis to assure the best and most efficient response to the ever-changing environment, under both normal physiological as well as pathological conditions. Transcriptional regulation of genes encoding enzymes and proteins involved in metabolic pathways represents one of the possible modes to control the flux of metabolic pathways. By increasing or reducing the transcription rate of genes encoding rate-limiting enzymes, it is possible to modulate metabolic pathways according to the cellular needs dictated by external stimuli. Gene transcription is a complex process requiring many components that regulate the reading of the genetic information, deconvolution to mRNA and subsequent translation to proteins. In eukaryotes, a complex machinery, constituted by dozens of factors physically or functionally associated with RNA polymerase II, transcribe genome regions encoding proteins involved in metabolic regulation. Such machinery is regulated by transcription factors, proteins binding specific DNA sequences located in proximity (promoters) or even at far distances from coding regions (enhancers), and their interacting cofactors or coregulators that either activate (coactivators) or repress (corepressors) gene transcription. Among the many transcriptional coactivators that have been identified so far, peroxisome proliferator activated receptor (PPAR) γ coactivator 1 α (PGC1α) has shown remarkable properties as it is involved in many cellular processes, most of them ascribable to regulation of metabolic pathways. PGC1α was first reported in 1998 by Puigserver et al. [6], who identified the coactivator in a yeast two-hybrid screen to find PPARγ-interacting proteins from mouse brown adipose tissue. Knutti et al. [7] also identified PGC1α as a coactivator of steroid receptors in a functional genetic screen in yeast and the distribution in human tissues was also reported [8,9]. Subsequently, other PGC1 related coactivators were identified [10,11,12]. These coactivators, named PGC1β, PGC1-related coactivator (PRC) and PGC1 related estrogen receptor coactivator (PERC) were initially involved in proliferative signals as coactivator of nuclear respiratory factor 1 (NRF1) [10], in the liver adaptation to fasting via the nuclear receptors PPARα and hepatocyte nuclear factor 4 (HNF4) [11], and in enhancing estrogen receptor α (ERα) transcriptional activity in different promoters [12]. Since then, many publications have dealt with numerous aspects of the biology of these coactivators and it has become clear that they orchestrate a greater number of cellular processes than previously estimated. 

In this review, we will discuss the basic biology of PGC1 proteins and how they regulate cellular processes involving metabolic pathways localized in the cytosol and in mitochondria. The possible therapeutical applications will also be examined.

## 2. Regulation of Cellular and Mitochondrial Metabolism by PGC1s

**Figure 1:**
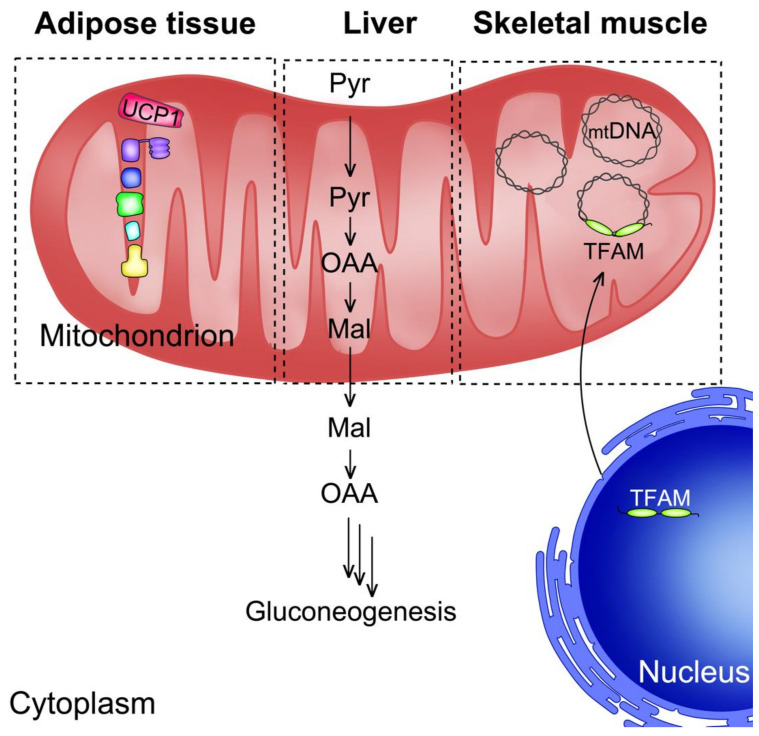
Roles of PGC1α in different tissues. A schematic summary of the roles of PGC1α in adipose tissue, liver and skeletal muscle discussed in this section is depicted. UCP1, uncoupling protein 1; Pyr, pyruvate; OAA, oxaloacetate; Mal, malate; mtDNA, mitochondrial DNA; TFAM, mitochondrial transcription factor A.

In this section, we will summarize the role of PGC1α in classical metabolic tissues by analyzing some examples in the liver, adipose tissues and skeletal muscles. The main points discussed in this section are depicted in Figure 1.

### 2.1. PGC1α and Mitochondrial Thermogenesis: Where the PGC1 Journey Started from

When it was first discovered, PGC1α was described as a “cold-inducible coactivator of nuclear receptors linked to adaptive thermogenesis”. Puigserver et al. [6] observed that PGC1α mRNA levels in brown fat and skeletal muscle increase in mice exposed to cold. The transcription activity of the nuclear receptors PPARγ and thyroid hormone receptor (TR) is strongly enhanced and, as a result, the transcription of the uncoupling protein 1 (UCP1) gene is induced. In addition, they reported that the expression of mitochondrial genes (e.g., COX-II) is also induced in adipocyte cultures with ectopic expression of PGC1α. Notably, since the amount of mitochondrial (mt) DNA increases, they proposed that PGC1α could stimulate factors involved in the transcription and replication of mtDNA. The other relevant observation was that the forced expression in white adipocytes elicits the expression of *Ucp1* and mitochondrial genes of the ETC along with increased content of mtDNA. These results suggest that this cold-inducible coactivator could also stimulate adaptive thermogenesis in adipose cells normally not involved in heat production, a process defined as the “browning” of white fat.

### 2.2. PGC1α: Beyond Adipose Tissue and Heat Production

Since its initial identification as a cold-inducible coactivator of adaptive thermogenesis responsive to the β-adrenergic/cyclic AMP (cAMP)/PKA axis in brown adipose tissue, the role of PGC1α was investigated in other tissues responsive to this signaling cascade. Two elegant studies demonstrated that PGC1α is a key coactivator for the transcription of the cytosolic phosphoenolpyruvate carboxykinase (PEPCK1, *PCK1*) and glucose-6-phosphatase (*GCPC*) genes, leading to increased glucose production and release by the liver in response to fasting. In the former study, the authors showed that the *PCK1* gene promoter is highly activated by PGC1α acting as a coactivator of HNF4α and the glucocorticoid receptor (GR), two important nuclear receptors required to actuate the response of the liver to fasting [13]. In the other study, it was found that the cAMP response element binding protein (CREB), a transcription factor activated by cAMP/PKA-dependent receptor cascades (e.g., β-adrenergic and glucagon signaling) activates the transcription of PGC1α gene (*PPARGC1A*) and of the downstream target genes *PCK1* and *GCPC*, consequently enhancing hepatic gluconeogenesis in the fasted state [14]. In a subsequent report, by using a dominant negative mutant of the forkhead transcription factor FOXO1, the same group demonstrated that PGC1α also interacts with and coactivates FOXO1 on the *PCK1* and *G6PC* genes, thus enhancing hepatic gluconeogenesis [15]. They also showed that insulin inhibited the PGC1α-FOXO1 interaction via Akt-mediated phosphorylation, thus providing a novel mechanistic explanation of the dominant suppressor effect of insulin on gluconeogenesis.

Remarkably, the hepatic expression of this coactivator is highly boosted under conditions resembling obesity and diabetes [13,15,16] and this leads to higher glucose production by the liver, which contributes to the development of type 2 diabetes. In obesity and diabetes, the liver and other organs experience endoplasmic reticulum (ER) stress, a condition whereby high levels of unfolded proteins are not sufficiently counterbalanced by proteins monitoring and assisting proper folding. The adaptive mechanism to ER stress is the unfolded protein response (UPR) that re-equilibrates proper ER homeostasis [17,18,19]. The transcription factor X-box binding protein 1 (XBP1), along with other transcription factors like ATF6, mediates the UPR and increases the folding capacity of ER proteins [20,21]. It has been documented that the restoration of XBP1 function in diabetic mouse models adjusts glucose homeostasis via the suppression of hepatic gluconeogenesis. This effect is mediated by the suppression of gluconeogenic genes via XBP1-mediated FOXO1 degradation [22,23,24]. In this regard, Lee et al. [25] made an unusual observation describing PGC1α as a co-suppressor of XBP1s in gluconeogenesis. In their in vitro and in vivo experimental models, they observed that PGC1α interacts with XBP1 and targets it to proteasomal degradation. They concluded that PGC1α does not only behave as a transcriptional coactivator of nuclear receptors but it can also suppress the activity of XBP1, a critical transcription factor mediating the UPR and the downregulation of the genetic program underlying gluconeogenesis. Thus, they proposed that therapeutic strategies interfering with PGC1α-XBP1 interaction may be envisaged to restore glucose homeostasis in obesity and diabetes. In this regard, a selective chemical inhibitor of PGC1α identified in a chemical screen has been shown to improve type 2 diabetes by increasing the acetylation and consequently reducing the HNF4/PGC1α gluconeogenic transcription program [26]. It would be interesting to verify whether this chemical inhibitor could also affect the interaction with XBP1, hence the capacity to suppress XBP1 activity.

### 2.3. PGC1α: Not Just Hot and Sweet Dreams

Since PGC1α is a coactivator of HNF4α, other investigators reasoned that it could regulate the expression of HNF4α-dependent genes relevant to additional metabolic pathways. In fact, independent groups discovered that PGC1α is also involved in the coordinated regulation of the *PCK1* gene and of the cholesterol 7α-hydroxylase gene (*CYP7A1*), the rate limiting enzyme of cholesterol conversion to bile acids [27,28]. The authors showed that physiological concentrations of chenodeoxycholic acid impair the protein-protein interaction between PGC1α and HNF4α, resulting in reduced transcription and expression of *CYP7A1* and *PCK1* genes in the liver [27]. They also showed that *CYP7A1* gene transcription is induced in parallel with *PCK1* in fasted mice, arguing that bile acid and glucose metabolism are coordinately regulated through the action of PGC1α on the gene transcription of the rate-limiting enzymes of these two metabolic pathways. Given the role of bile acids in suppressing the transcription of these two genes responsive to nutritional status, they proposed that bile acids are important regulators of cholesterol and glucose metabolism in the fasted-to-fed cycle. These studies underline PGC1α as a central hormone-sensitive regulator of metabolic pathways.

### 2.4. PGC1α: Into the Power Plant

Several studies indicate PGC1α as one of the key regulators in mitochondrial biology. As previously mentioned, higher expression of PGC1α in adipocytes increases mtDNA content and the expression of genes of the electron transport chain [6], suggesting that this coactivator could orchestrate mitochondrial biogenesis and metabolism by inducing the transcription and replication of mtDNA. Based on these observations, it was hypothesized that this paradigm could be applicable to other cell types and tissues. Indeed, forced expression of PGC1α in myotubes increases oxygen consumption and the expression of OxPHOS genes [29]. In the same study, the authors showed that the overexpression of PGC1α in myotubes leads to overt mitochondria proliferation as assessed by electron microscopy. The molecular mechanism underlying mitochondrial biogenesis involves a PGC1α-dependent activation of the transcription of nuclear respiratory factor (NRF) 1 and 2. These TFs are known to induce the expression of genes in ETC, along with the mitochondrial transcription factor A (mtTFA or TFAM), a nuclear factor regulating the transcription and replication of mtDNA [30,31]. 

In line with these observations, transgenic mice overexpressing PGC1α, specifically in skeletal muscles via the muscle creatine kinase promoter, switch glycolytic type II fibers to type I fibers, which are characterized by a higher mitochondrial number, oxidative metabolism and express genes typical of type I fibers like troponin I (slow) and myoglobin [32]. The mechanism underlying the described phenotype of this transgenic mouse model indicates that PGC1α coordinates the expression of genes involved in oxidative metabolism via the transcription factor NRF1, and the expression of genes of skeletal muscle type I fibrillar proteins via calcineurin-dependent coactivation of MEF2 transcription factors. Overall, these observations provide evidence of the central role of PGC1α in metabolic and fiber type switch in skeletal muscles, adjusting the metabolic requirements and muscle type fibers in response to environmental cues that induce PGC1α activity and expression.

In a more recent study, Koh et al. [33] found out that the pyruvate mitochondrial transporter MPC1 is a target of PGC1α and drives the mitochondrial respiratory capacity. The authors of this study reported that MPC1 and PGC1α expression is downregulated in renal cell carcinoma (RCC). Overexpression of PGC1α in RCC cell lines restores MPC1 expression and this effect requires the nuclear receptor estrogen-related receptor α(ERRα). Most importantly, MPC1 is strictly indispensable to drive mitochondrial biogenesis, as inactivation of *MPC1* by CRISPR/Cas9 mutagenesis impairs the PGC1α-mediated increase of mtDNA in RCC cells. Globally, these results underline the key role played by MPC1 in pyruvate-dependent OxPHOS and mitochondrial biogenesis induced by PGC1α. Of note, we also reported a critical role of MPC1 in thermogenesis, as the expression of *Mpc1* increases in histone deacetylase 3 (HDAC3) knockout mice in adipose tissue [34]. This mouse model displays a peculiar metabolic rewiring that sustains the browning of white fat. More specifically, we observed a futile cycle of fatty acid metabolism whereby de novo synthesis and β-oxidation of fatty acids occur concomitantly to match the high energy demand of thermogenic adipocytes. In this context, the elevated expression of *MPC1* is coupled with that of other important genes of glucose metabolism and tricarboxylic acid (TCA) cycle. MPC1 provides pyruvate to the anaplerotic enzyme pyruvate carboxylase, which converts it to oxaloacetate required to condensate with acetyl-CoA derived from fatty β-oxidation and to initiate de novo fatty acid synthesis in the cytosol. In addition, since the expression of *PCK1* also increases in the adipose tissue of HDAC3 knockout mice, we speculated that oxaloacetate could also feed another futile cycle of phosphoenolpyruvate, pyruvate and oxaloacetate itself to provide ATP in the cytosol necessary to allow the conjugation of de novo synthesized fatty acids with CoA and the subsequent transport into the mitochondrial matrix for β-oxidation. The importance of *MPC1* expression in thermogenesis is also supported by the higher mRNA levels in BAT [34]. Last but not less important, fat-specific HDAC3 KO mice also show increased mRNA level of PGC1α as assessed by RNA-seq, once again linking increased mitochondrial metabolism to PGC1α-mediated transcriptional regulation.

## 3. Regulation of PGC1s

Regulation of PGC1s primarily relies on changes in gene expression in response to different stimuli (e.g., cold exposure, fasting). All members of PGC1 family act as coactivators of mitochondrial biogenesis although they present different expression pattern in distinct cell types. Such variation reflects specialized functions for PGC1α, PGC1β and PRC alongside their redundant role in supporting energy metabolism.

Post-translational modifications (PTMs) have a role in the rapid regulation of protein activity. In this context, several PTMs have been described for PGC1α including phosphorylation, methylation and acetylation. One of the first PTMs discovered for PGC1α was the phosphorylation by p38 MAPK upon cytokine stimulation. p38 MAPK is responsible for phosphorylation of different transcription factors (e.g., ATF-2, MEF-2, Elk1) and directly phosphorylates PGC1α at three residues: Thr262, Ser265 and Thr298. These modifications increase the protein levels by extending its half-life and are responsible for elevated oxygen consumption rates in cultured myotubes; they are therefore considered activation marks of PGC1α activity. Cytokines are able to act via the p38 MAPK pathway and in fact activate PGC1α, thus promoting gene transcription of downstream targets [35]. Afterwards, another study performed by the same group shed light on the mechanism responsible for this modulation; p38 MAPK-related phosphorylations occur in the suppression domain of PGC1α (aa 200–400) and prevent binding of p160^MBP^, a transcriptional suppressor. When PGC1α is not phosphorylated, it can dock to promoters but does not positively modulate transcription of target genes because of p160^MBP^ inhibition [36], thus clarifying previous observations made by Knutti et al. [37]. Subsequently, Crunkhorn et al. [38] highlighted the importance of p38 MAPK-mediated regulation of PGC1s in the context of obesity. They firstly demonstrated that mRNA levels of PGC1α and β decrease in the quadriceps muscle of C57Bl/6 mice after high fat diet (HFD) feeding. In particular, in vitro studies on myotubes disclosed that this effect is mediated by long chain saturated fatty acids, specifically palmitate and stearate. Palmitate treatment induces phosphorylation of p38 MAPK so that regulation of PGC1s occurs via modulation of p38 activity. In contrast to previous evidence, in this case a sustained p38 MAPK activity is linked with a reduction in PGC1s levels and consequent lower expression of TCA cycle and oxidative phosphorylation genes. However, diminished expression of PGC1s can be caused by the effect of palmitate on promoter activity. In fact, palmitate seems to act also via histone deacetylation because cotreatment with trichostatin A, an inhibitor of class I and II histone deacetylases, recovered PGC1α expression.

Furthermore, PGC1α can be phosphorylated by glycogen synthase kinase 3β (GSK3β) at Thr265 when it is primed with p38 MAPK phosphorylation. Interestingly, this active form of PGC1α can be tagged for proteasomal degradation by E3 ubiquitin ligase SCF^Cdc4^. Ubiquitination occurs after p38 MAPK and GSK3β phosphorylation because SCF^Cdc4^ recognizes a phosphothreonine-containing motif, known as Cdc4 phosphodegron (CPD), for its activity. Intriguingly, in neurons levels of SCF^Cdc4^ are reduced in oxidative stress conditions, resulting in increased levels of activated PGC1α. In this setting, PGC1α, as well as enhancing oxidative metabolism, promotes transcription of antioxidant genes (e.g., catalase, superoxide dismutase, glutathione peroxidase, and thioredoxin 2) to protect cells from ROS damage [39]. Other kinases directly phosphorylate PGC1α. Jäger et al. [40] demonstrated that AMP-activated protein kinase (AMPK) phosphorylates PGC1α at Thr177 and Ser538, leading to its activation and further promoting its transcription in a feed-forward loop in skeletal muscle. By studying myotubes from mice with tissue specific knockout of PGC1α, they assessed that most of the positive effects of AMPK activation on glucose uptake and mitochondrial functionality are mediated by increased activity of PGC1α. Moreover, the induction of other genes, such as *Ucp3* and *Pdk4*, is PGC1α-independent. AMPK-dependent activation of mitochondrial biogenesis and functionality can occur upon feeding with high dietary polyunsaturated fatty acids (PUFAs) [41], with the administration of the antidiabetic drug liraglutide [42] and treatment of 3T3-L1 preadipocytes with magnesium and calcium-enriched deep-sea water [43]. In addition, AMPK activation followed by increased PGC1α expression in skeletal muscle was observed upon central administration of leptin and switches on PI3K pathway in the hypothalamus [44]. However, activation of AMPK is not necessarily linked to PGC1α-dependent mitochondrial biogenesis, as demonstrated in the context of lipopolysaccharide (LPS)-chronic mild stress model [45]. In addition, other studies on hepatic cells revealed that phosphorylation of Ser570 of PGC1α is mediated by Akt/PKB in response to insulin signaling. In the liver, this PTM is crucial to regulate glucose homeostasis; in particular, Akt-dependent phosphorylation results in inhibition of PGC1α by displacing it from chromatin and preventing transcriptional coactivation [46]. PGC1α plays a pivotal role in controlling processes from fasting to fed state and needs to be strictly regulated. S6 kinase 1 (S6K1), a serine/threonine kinase modulated by insulin and nutrients, phosphorylates PGC1α on Ser568 and Ser572. These PTMs do not affect mRNA or protein levels but attenuate PGC1α positive control of gluconeogenesis with no effects on other pathways. The precise regulation of PGC1α activity is possible via blocking its binding to specific factors; in this case, phosphorylation by S6K1 prevents the interaction with HNF4α, a nuclear receptor that mediates the induction of gluconeogenic genes as discussed above. Other activities of PGC1α, including FA β-oxidation and ETC, are spared from S6K1 regulation so that the liver can rely on FA catabolism for its functions even after a high-carbohydrate but low-calorie meal [47].

Moreover, PGC1α ability to coactivate HNF4α is reduced by acetylation, which occurs at 13 lysine residues. In mouse liver, fasting increases PGC1α levels alongside SIRT1, a NAD^+^-dependent class III histone deacetylase. SIRT1 is regulated by nutritional status and in fact (1) SIRT1 protein synthesis is boosted by pyruvate and (2) NAD^+^ levels increase during fasting and return to basal levels after refeeding. Interestingly, in hepatocytes SIRT1 is present in a complex with PGC1α and HNF4α, which control in opposite ways gluconeogenesis (inducing *PEPCK* and *G6Pase*) and glycolysis (decreasing glucokinase and liver pyruvate kinase) depending on pyruvate levels. The complex activation results in increased glucose production under nutrient deprivation [48]. On the contrary, when PGC1α is in complexes containing the acetyltransferase GCN5, gluconeogenic activity is repressed. GCN5 regulation, which antagonizes SIRT1 activity, occurs by displacing PGC1α from active chromatin and confining it in repressive nuclear foci [49].

PGC1α can also be positively modulated via methylation of Arg665, Arg667 and Arg669 by protein arginine methyltransferase (PRMT1), which acts synergistically on transcription. Methylation occurs in a region with a subnuclear localization signal, therefore it modulates PGC1α nuclear distribution or elicits a conformational change enhancing the binding with coactivator complexes [50]. Nevertheless, Qian et al. [51] recently demonstrated that monomethylation of Lys224 antagonizes PGC1α positive regulation of mitochondrial biogenesis. This PTM is particularly relevant in the modulation of PGC1α activity depending on oxygen availability. In fact, lysine demethylase 3A (KDM3A), which relies on oxygen for its activity, demethylates PGC1α at this residue. In hypoxic condition KDM3A is inhibited and monomethylated PGC1α increases resulting in reduced mitochondrial function. Oxygen supply is critical in solid tumor growth where abrogation of oxidative metabolism protects cancerous cells from ROS damage and a switch to glycolytic metabolism provides carbon intermediates for biosynthetic processes. By expressing the PGC1α K224R mutant, authors were able to prevent inactivation due to monomethylation in hypoxic conditions, thus resulting in increased tumor cell apoptosis and inhibition of tumor growth.

A recent study on PGC1β revealed that regulation of this coactivator family occurs also at a post-transcriptional level. In cardiomyocytes, PGC1β expression is downregulated by miR-30c, a miRNA whose abundance is reduced by diabetes. Reduction of miR-30c eventually leads to lipotoxicity and reduced glucose utilization via PGC1β-dependent PPARα transcriptional activation. Overexpression of miR-30c reduces myocardial lipid accumulation and attenuates cardiomyocytes apoptosis, ameliorating cardiac dysfunction [52]. On the other hand, PGC1β overexpression may be a tool to partially counteract obesity. In this perspective, Uchitomi and Nakai screened food compounds that induce PGC1β and disclosed that soy isoflavones (genistein and daidzein), and resveratrol activate ERR-mediated transcription via PGC1β in myotubes, ultimately increasing expression of the target gene *Acadm* [53,54].

To sum up, the regulation of members of the PGC1 family occurs at different levels: transcriptional, post-transcriptional, and post-translational. Since mitochondrial biogenesis and functionality are implied in several physiological processes in most organs and tissues, knowledge about regulation of PGC1 family members is essential to develop new tools for treating and ameliorating a plethora of diseases.

## 4. Role of Mitochondria and PGC1 Proteins in the Modulation of the Immune System

Recently, it has become evident that metabolic adaptation and mitochondrial functions are central to several aspects of immune cells. The complexity of the immune system in terms of origin, cell types and subpopulation, responses to stressors and roles in various diseases, is paralleled by a variety of metabolic phenotypes. Readers interested in specific and in-depth analysis of these aspects may refer to recently published review articles [55,56,57,58,59,60].

In this section, we will briefly summarize recent evidence highlighting the role of mitochondria and members of the PCG1 family in the immune system.

The changes in mitochondria dynamics, biogenesis and activity, which affect most, if not all, the features and stages of immune cells, are key concepts to understand and interpret the studies in the field. The most relevant stages and processes in immune cell biology are: proliferation, differentiation, activation and polarization, response to pathogens or other systemic and microenvironmental cues, cellular fate (including exhaustion), and death. Thus, mitochondrial regulators such as PGC1 coactivators, TFAM and factors involved in mitochondrial fusion and fission are expected to play a central role in the abovementioned pathways and states.

Before discussing specific studies, we will recap some biochemical key points to fully appreciate how the multifaceted functions of the innate and adaptive immune cells are intertwined with mitochondria biology and metabolism.

- Glycolytic flux ending with the formation of lactate in normoxic conditions (the so-called aerobic glycolysis or the Warburg effect): it produces ATP independently of mitochondria; the amount of ATP produced by this pathway can contribute to support the cellular needs and functions; upregulation of lactate dehydrogenase isoform A (LDHA) is responsible for the conversion of the glycolytic end-product pyruvate to lactate, acting either as metabolic substrate and/or signaling molecule.
- Catabolic TCA cycle: it is fueled by acetyl-CoA derived either from pyruvate, the end-product of glycolysis, or from fatty acid oxidation. In normoxic conditions, catabolic TCA cycle is coupled with ETC-OxPHOS; here the reducing equivalents (NADH and FADH_2_) produced by oxidative reactions are re-oxidized generating the electron flow and proton pumping necessary for mitochondrial ATP synthesis. High rates of oxidative metabolism and electron transfer at the inner mitochondrial membrane may be responsible for ROS formation and accumulation if they are not inactivated by efficient antioxidant systems.
- Anabolic TCA cycle: glutamine-derived α-ketoglutarate enters the TCA cycle and provides intermediates that can be diverted from the cycle to produce aspartate (deriving from oxaloacetate), a building block for nucleotide synthesis, or acetyl-CoA moieties (deriving from citrate) used for lipid synthesis; anabolic TCA is especially active in proliferating cells.
- Mitochondrial damage-associated molecular patterns (MDAMPs): mitochondrial derived material is released either in the cytoplasm by dysfunctional organelles or in the extracellular space upon cell death; it includes mtDNA, ATP, TFAM, N-formyl peptides, succinate (a TCA cycle intermediate), and cardiolipin (a phospholipid highly enriched in the inner mitochondrial membrane) and induces inflammatory responses similar to those elicited by pathogen-associated molecular patterns (PAMPs).

### 4.1. Mitochondria in Immune Cells

**Figure 2:**
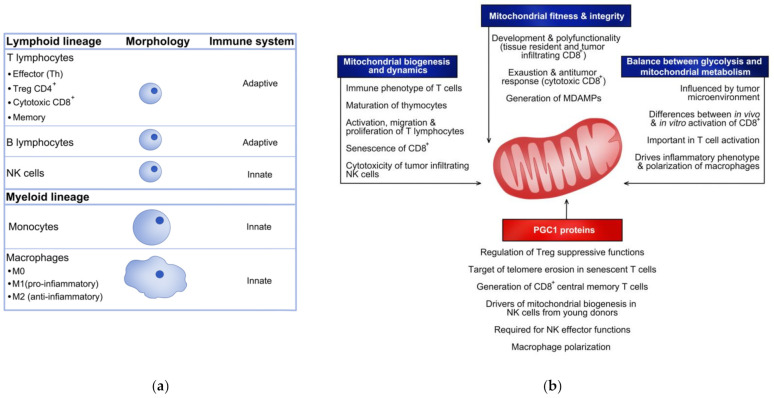
Role of mitochondria and PGC1 proteins in the immune system. Schematic list of the immune cell populations cited in the text (**a**). Summary of functions and phenotypes of different immune cells affected by mitochondrial features and PGC1 proteins (**b**).

Immune cells can be distinguished according to their lineage (lymphoid or myeloid lineage) or whether they are part of the innate of adaptive arm of the system. A schematic representation of the immune cells that will be discussed below is reported in Figure 2a.

#### 4.1.1. Mitochodrial Mass, Dynamics and Biogenesis Impact Functions and Fates of Immune Cells

Mitochondria fusion and fission and the proteins that orchestrate these processes have been thoroughly investigated in T cells in relation to the different subpopulations and during the development and activation state of these lymphocytes.

A hallmark study published in 2016 by the group of Erika Pearce [61] investigated in depth the morphology and dynamics of mitochondria in different T cells subtypes; the authors established a causative correlation between mitochondrial morphology, the metabolic phenotype, and the immune features of some T lymphocytes subtypes. They discovered that activated effector T cells are characterized by intense anabolism and punctate mitochondria; on the other hand, memory T cells, exhibiting a prevalent catabolic phenotype, present fused mitochondrial networks. The mitochondria-shaping protein optic atrophy 1 (OPA1) was found to be fundamental to maintain fused morphology and shape cristae to favor OxPHOS and fatty acid oxidation in memory T cells. These observations provided evidence that changes in mitochondria morphology impose metabolic reprogramming in T cells, thus controlling their fate toward a specific immune phenotype.

The lymphoid precursor cells migrate from the bone marrow to the thymus where they develop to different T lymphocytes starting from a common initial stage of thymocytes. Therefore, maturation in the thymus represents a key step in the developmental program of T cells. It has been recently discovered that deletion of OPA1 in thymocytes at early stages impairs respiration and favors cell death, thus compromising the progress of the developmental program [62]. Moreover, the thymocytes that succeed in exiting the thymus despite OPA1 deficiency exhibit an effector memory phenotype but do not generate functional long-term memory T cells. These findings indicate that perturbating mitochondrial dynamics and, consequently, impairing mitochondrial functions at early stages dramatically impact the immune phenotype of mature T cells.

The GTPase Dynamin-1-like protein (DRP1) is a well-established key component of the mitochondrial fission machinery [63,64]. This protein has been recently discovered to sustain both migration and expansion of T cell precursors residing in the thymus. It has been demonstrated that the presence of DRP1 is implicated in diverse aspects of developing T cells: metabolic rewiring following activation, proliferation by clonal expansion, migration and recirculation in secondary lymphoid organs [65].

Lymphoid precursors develop into different subtypes, among which are CD4 expressing (CD4^+^) T lymphocytes. Once naïve CD4^+^ T cells are exposed to antigens, they become activated and undergo proliferation. While naïve CD4^+^ T cells mainly rely on OxPHOS for ATP production, activated proliferating CD4^+^ T cells are characterized by aerobic glycolysis and utilization of mitochondrially derived substrates for biomass and cytokine production. However, hyperproliferation should be avoided, thus homeostatic systems favoring naïve CD4^+^ T cell quiescence are necessary to counteract the risk of CD4^+^ T cell overproduction. Lymphocyte activation gene-3 (LAG-3) has emerged as an important factor contributing to dampening proliferation and Interferon γ production in naïve CD4^+^ T cells. Previte et al. [66] investigated how LAG-3 modulates the metabolic phenotype of naïve CD4^+^ T cells. By analyzing CD4^+^ T cells from *Lag3*^−/−^ mice they discovered that this factor negatively regulates mitochondrial biogenesis, limiting oxygen consumption and spare respiratory capacity, thus favoring a quiescent state.

A detailed analysis of the metabolic profile and mitochondria remodeling during prolonged T cell activation revealed that mitochondria contribute minimally to ATP production [67]; in contrast, they are crucial for the generation of ROS acting as intracellular signaling mediators. Of note, during T cell activation the authors of the study also observed increased mitochondrial content and volume and mitochondrial biogenesis. These findings indicate that remodeling of mitochondria morphology and mitochondria biogenesis are important for the cellular functions of activated T lymphocytes, independently from their role in ATP production.

CD4^+^ and CD8^+^ T lymphocytes display distinctive features, such as immune phenotype, immune response, cell fate. Callender et al. [68] have recently reported that differences in the mitochondrial mass drive the susceptibility of human CD8^+^ T cells to senesce faster than CD4^+^ T cells. In fact, the greater mitochondrial mass and higher metabolic flexibility observed in senescent CD4^+^ T cells with respect to senescent CD8^+^ T cells translate into a metabolic advantage. Consequently, senescent CD4^+^ T cells proliferate and migrate more than senescent CD8^+^ T cells. These findings may contribute to uncovering the mechanistic links between mitochondrial (dys)function and cellular senescence and aging.

Mitochondria morphology and dynamics also play a role in natural killer (NK) cells and affect their function of tumor surveillance. In this regard, it has been reported that tumor-infiltrating NK cells display fragmented mitochondria in contrast to NK cells present in non-tumor compartments, which are characterized by normal large, tubular mitochondria [69]. The fragmented morphology correlates with reduced cytotoxicity and increased ability of the cancer cells to evade the NK cell-mediated surveillance. The mechanism underlying mitochondrial fission and fragmentation in tumor infiltrating NK cells is activation of an mTOR-Drp1 axis due to the hypoxic tumor microenvironment. These findings suggest that prevention of mitochondrial fragmentation may help to maintain the antitumor activities of NK cells.

#### 4.1.2. Mitochondrial Fitness and Integrity in Immune Cells

Metabolic and mitochondrial fitness are essential to preserve cellular functions. To maintain cellular well-being, dysfunctional organelles, such as depolarized mitochondria that lead to apoptosis and cell death, are degraded through mitophagy. Recent studies have revealed that mitochondrial fitness plays a key role in T cell functions and fate.

Li et al. [70] investigated the transcription factor BHLHE40 in tissue-resident memory CD8^+^ T cells and tumor-infiltrating lymphocytes, which are involved in antiviral and antitumor responses, respectively. Interestingly, the authors found that the mitochondrion is the primary target of Bhlhe40-directed gene expression since this factor positively regulates the expression of genes encoding the components of the mitochondrial membrane or genes involved in mitochondrial metabolism and/or OxPHOS. The authors also found that Bhlhe40-dependent metabolic fitness is strongly associated with a functional epigenetic state required for the development and polyfunctionality of tissue resident and tumor infiltrating lymphocytes. In summary, the mechanism responsible for programming the mitochondrial and epigenetic regulation of resident CD8^+^ T cell functionality in situ identified in this study is critically important in the antiviral and antitumor activities of these cells.

Mitochondrial fitness, and its impact on antitumor activity in tumor-infiltrating T lymphocytes, has also been investigated by Yu et al. [71]. The authors found that microenvironmental cues and other signals lead to accumulation of depolarized mitochondria, as a result of reduced mitophagy. Loss of mitochondrial and metabolic fitness ultimately leads to the so-called exhaustion, a dysfunctional state of cytotoxic CD8^+^ T cells, usually observed in chronic infection and cancer. This study demonstrates that the T cell antitumor responses are mechanistically linked to mitochondria dynamics and quality.

Another important issue is that mitochondrial integrity of immune cells is essential to prevent the release of mitochondrial damage-associated molecular patterns such as mtDNA. The presence of mtDNA in the cytoplasm is detected by the cyclic GMP-AMP synthase-stimulator of interferon genes (cGAS–STING) pathway, which in turn activates defensive responses that vary depending on the cell type. In this context, Field et al. [72] recently discovered that mitochondrial integrity and functions of regulatory T cells require the fatty acid binding protein FABP5. Pharmacological or genetic inhibition of this protein cause mitochondrial defects, i.e., dysregulation of the mitochondrial network, loss of ETC complexes, and decreased mitochondrial OxPHOS. The mitochondrial and metabolic dysregulation ultimately increases the regulatory T cell suppressive capacity, enhances IL-10 production and suppression of proliferation in vitro. Interestingly, the mitochondrial defects could be associated with altered levels of cardiolipin, which is highly abundant in the inner mitochondrial membrane. In conclusion, this study highlights the strict links between lipid metabolism, mitochondrial integrity, and immune functions of regulatory T cells.

#### 4.1.3. New Insights on the Balance between Glucose and Mitochondrial Metabolism in Immune Cells

The metabolic setting required to maintain a quiescent state, the switch from OxPHOS to aerobic glycolysis that occurs during activation and proliferation, has been investigated in depth in T cells, B cells, and monocytes-macrophages [56]. In this regard, it should be underlined that (i) most of the studies have been performed using in vitro activated cells, and (ii) different stimuli/microenvironments impose different metabolic rewiring. In this section, we will only discuss the latest studies that by advanced technologies have added new insights or provided a deeper understanding of the issue.

Ma et al. [73] applied stable isotope tracing to gain insights on how T cells use metabolic substrates during immune responses following Listeria infection in vivo. The authors found that physiologically activated CD8^+^ T cells display greater rates of oxidative metabolism and higher bioenergetic capacity than in vitro activated CD8^+^ T lymphocytes. Moreover, the metabolic fate of glucose in CD8^+^ T cells changed dynamically over the course of an immune response. Glucose was found to sustain anabolic pathways, specifically de novo serine biosynthesis, thus allowing optimal T cell expansion in vivo. These findings underline the importance of the immune microenvironment in vivo that drives the metabolic phenotype of T cells during an active immune response.

Bailis et al. [74] investigated whether mitochondrial metabolism supports differentiation and effector function of mouse T helper 1 cells by distinct modes and independently. The authors found that the activity of succinate dehydrogenase, an enzyme complex that bridges TCA cycle and ETC, is required for terminal effector function of T helper 1 cells while it represses proliferation. On the other side, the activity of complex I of ETC, the malate-aspartate shuttle and the mitochondrial citrate export, supply substrates for anabolic pathways and for histone acetylation during T cell activation. Thus, the study unveiled that two key processes in T cells, i.e., proliferation and effector activity, are linked to mitochondrial metabolism but biochemically uncoupled.

The balance between glucose and mitochondrial oxidative metabolism has been investigated in macrophages as well [55]. In brief, proinflammatory macrophages (M1) are subject to increased rate of glycolysis upon activation, which is mediated by Hypoxia Inducible Factor 1α (HIF-1α) signaling; in parallel, these cells are characterized by an impaired TCA cycle, ETC and OxPHOS. Of note, a dysfunctional TCA cycle in activated inflammatory macrophages leads to the accumulation of intermediates or derivatives with important signaling functions; we refer specifically to succinate that contributes to the stabilization of HIF-1α, and therefore to the Warburg effect, and to itaconate, produced from aconitate through a diversion of the TCA cycle [75].

Interestingly, mitochondrial metabolism can be pharmacologically modulated (i) to induce macrophage re-polarization from M2-like phenotype with immunosuppressive functions that are detrimental in tumors, to M1-like proinfammatory antitumor phenotype; (ii) to prevent M0-to-M2 polarization. In this respect, Oyarce et al. [76] tested clinically applicable drugs targeting M2-specific metabolic pathways: fatty acid oxidation, glutaminolysis, PPAR activation, and mitochondrial ETC. For example, pharmacological inhibition of fatty acid oxidation or ETC, impaired mitochondrial basal, and maximal respiration, resulted in M2 to M1-like re-polarization and strong tumor-cytotoxic activity. Therefore, the pharmacological modulation of mitochondrial metabolism is an efficient tool to modulate the macrophage phenotype with important consequences in the tumor microenvironment. A summary of the concepts discussed above is depicted in Figure 2b.

### 4.2. PGC1 Proteins in Immune Cells

It is generally assumed that the mitochondrial response to stressors (including sepsis) is orchestrated by PGC1α. Despite the wealth of studies addressing the role of mitochondria in different immune cells, the direct involvement of members of the PGC1 family has been less investigated directly or only presumed. In this section, we will discuss some of the most recent studies that specifically investigated the role of PGC1 proteins in different immune cells. Figure 2b illustrates the main functions of PGC1 proteins in immune cells that will be discussed below.

#### 4.2.1. PGC1 Proteins in Cells of the Lymphoid Lineage

In a study published in 2015, Beier et al. [77] investigated mitochondrial functions in conventional T cells with respect to regulatory T cells. Based on the premise that Sirt3 and PGC1α are essential for physiologic mitochondrial activity, the authors analyzed the metabolic and immune phenotype of T cells isolated from mice lacking these two factors. The authors found that loss of Sirt3 or PGC1α weakens Treg suppressive functions, confirming the strict dependence of this T cell subtype upon OxPHOS. In contrast, the authors observed that genetic modulation leading to increased Treg suppressive function, e.g., HDAC9 deletion, is associated with higher expression of Sirt3 and PGC1α and improved mitochondrial respiration. Therefore, this study established the direct involvement of PGC1α, especially in regulatory T cells in which the suppressive phenotype is strictly linked to OxPHOS.

More recently, mitochondrial (dys)regulation and the role of PGC1α were investigated in the context of T cell senescence and apoptosis linked to telomere erosion. Schank et al. [78] demonstrated that telomere injury causes generalized mitochondrial dysfunction, i.e., swelling, decreased membrane potential, reduced OxPHOS activity, respiration rate and mtDNA content, etc. At the mechanistic level, the authors discovered a regulatory axis by which telomere injury activates p53 signaling, which is in turn responsible for repression of PGC1α and nuclear respiratory factor 1 (NRF1). Thus, telomere loss occurring during aging could be a driving cause of mitochondrial dysfunction by targeting two master mitochondrial regulators, PGC1α and NRF1.

Dumauthioz et al. [79] studied the role of PGC1α in memory CD8^+^ cells and tumor-infiltrating lymphocytes, two subpopulations characterized by different metabolic settings. In memory CD8^+^ cells OxPHOS and fatty acid oxidation are crucial to maintain proliferative capacity, self-renewal, and long-term protection against tumors; as already mentioned, in the tumor microenvironment tumor-infiltrating lymphocytes undergo mitochondrial dysfunction and, ultimately exhaustion. The authors of the study found that overexpression of PGC1α favors the generation of central memory CD8^+^ T cells rather than the formation of resident memory cells. Furthermore, PGC1α-overexpressing CD8^+^ T cells mediate a more robust response to infection and vaccination and provide a stronger antitumor immunity. Based on these observations, it can be concluded that favoring mitochondrial biogenesis by enforced PGC1α expression promotes cellular fitness and functions in memory CD8^+^ cells.

When discussing the role of PGC1α in NK cells, it should be mentioned that the cytotoxic activity of this subpopulation of lymphocytes is modulated by the cytokines they are exposed to and is linked to the metabolic setting. Miranda et al. [80] investigated the mitochondrial phenotype of human NK cells exposed to IL-2, known to induce a more potent cytotoxic activity. The authors found that IL-2 mediated activation is associated with mitochondrial biogenesis and increased mitochondrial membrane potential and these effects rely on PGC1α, as demonstrated by gene silencing. Interestingly, the authors also found that inhibition of ATP synthase with oligomycin dampens the cytotoxic activity. The same group investigated the metabolic and immune profile of human NK cells exposed to IL-2 in relation to the age of the donor. The rationale of the study was that human NK cells display aging-related dysfunction, a feature associated with higher tumor incidence, reduced vaccination efficacy and poor response to infections in the elderly. The authors found that only in NK cells isolated from young subjects, exposure to IL-2 elicited an improved mitochondrial phenotype, which correlated with increased expression of PGC1α; on the contrary, in NK cells from elderly donors PGC1α expression was reduced and ROS formation increased in response to IL-2 [81].

More recently, Gerbec et al. [82] found that in vivo activation of NK cells upon Listeria monocytogenes infection leads to increased expression of PGC1α target genes. The authors demonstrated that conditional deletion of PGC1α in NK cells impairs the effector functions, bioenergetics, and cytotoxic potential of these cells. Furthermore, the lack of PGC1α in NK cells in an experimental model of melanoma, negatively affects the transcription of genes required for mitochondrial activity; these observations further underline the functional relevance of mitochondria for optimal NK cell functions.

#### 4.2.2. PGC1 Proteins in Cells of the Myeloid Lineage

The role and regulation of PGC1 proteins in monocytes and macrophages are strictly related to the functions of these cells in inflammatory diseases or conditions.

In general, inflammatory cues such as tumor necrosis factor, interleukin-1β, and lipopolysaccharide downregulate PGC1α expression in different tissues and cell types, an effect that contributes to the inflammatory state [83].

An inverse correlation has been found between PGC1α expression and the inflammatory state of cells and organs; this is because in most compartments PGC1α positively regulates the expression of antioxidant genes, thus reducing the levels of ROS and their proinflammatory signaling. Therefore, it is not surprising that PGC1α participates in macrophage polarization promoting transition from M1-like proinflammatory phenotype to M2-like anti-inflammatory phenotype.

While the overall effects of PGC1α deficiency in different organs and tissues have been addressed in several studies, especially in relation to cardiovascular disease and atherosclerosis [84], the specific roles of PGC1 proteins in monocyte-macrophages remain less defined.

With respect to PGC1β, it was demonstrated that direct genetic manipulation of PGC1β, either silencing or overexpression, in macrophages significantly impacts mitochondrial oxidative program and the anti-inflammatory program. In line with its known role, PGC1β overexpression in macrophages induces mitochondrial biogenesis and oxidative metabolism and inhibits the production of proinflammatory cytokines [85].

In conclusion, in this section we selected and discussed recent examples underlying the importance of mitochondria in many immune cell populations. Even though in most cases the implication of PGC1 proteins has not been directly addressed or demonstrated, it is reasonable to hypothesize that they play a pivotal role in modulating processes such as biogenesis and oxidative metabolism profoundly affecting immune cells’ development and functions.

## 5. Beyond PGC1s in the Regulation of Cellular and Mitochondrial Metabolism

Current knowledge indicates that the inducible process of mitochondrial biogenesis is under the control of the nuclear and mitochondrial compartments and must be tightly and reciprocally controlled to fine-tune mitochondrial mass and functions in response to extra and/or intracellular signals to adapt to certain energy needs [1,86,87,88]. Examples are represented by the mitochondrial biogenesis induced by calorie restriction or that observed in skeletal muscle during exercise training [89,90]. In addition, the bigenomic origination means that mutations in either mitochondrial or nuclear genes can produce mitochondrial dysfunction leading to the development of mitochondrial disorders [91,92].

### 5.1. Screening Strategies to Identify Novel Mitochondrial Regulators

Since its discovery, PGC1α has been considered the master regulator of mitochondrial biogenesis [29,87,88,93], however several other regulators acting on mitochondrial function together with mitochondrial quality control systems (fission/fusion and mitophagy) [94] fine-tune and maintain mitochondrial homeostasis. Considering that (i) mitochondria are complex organelles where many pathways and cell functions overlap, (ii) the crosstalk with other organelles contributes to the complexity, (iii) signals or states resulting in mitochondrial dysfunction are still poorly understood and (iv) the lack of curative therapies for mitochondrial dysfunction and diseases, the known mitochondrial regulators still do not exhaustively describe the complexity of the circuits governing mitochondrial functions. Therefore, in the last two decades several research groups have performed high throughput screening studies to identify novel mitochondrial regulators relevant in physiology and pathophysiology.

As already mentioned, PGC1α itself was originally discovered in a yeast two-hybrid system-based screen, with the aim to identify PPARγ-interacting proteins from murine brown fat cells [6]. Another key regulator of mitochondrial biology, PR/SET Domain 16 (PRDM16) was also isolated with a global expression screen with the goal of identifying transcriptional components that regulate the development and function of brown adipocytes [95]. These pioneering screens clearly demonstrated the ability of isolating novel mitochondrial regulators. Chen and colleagues [96] performed a genome-wide RNA interference (RNAi) screen in *Drosophila* cells using mitochondrial citrate synthase (CS) activity as the primary readout and identified 152 genes as modulator of CS activity. A few years later Gohil and coworkers [97] performed a nutrient-sensitized screen searching small molecules to selectively impair growth and viability of human fibroblasts in media containing either galactose or glucose as the sole sugar source. Several clinically used drugs, among which was meclizine, never associated with energy metabolism attenuate mitochondrial respiration through a mechanism distinct from that of canonical inhibitors. The same laboratory used a genome wide CRISPR “death screen” that actively selects dying cells to reveal human genes required for OxPHOS, inspired by the classic observation that human OxPHOS-deficient cells survive in a medium containing glucose while they die in the presence of galactose. This screen unraveled a functional network involved in the regulation of the mitochondrial 16S ribosomal RNA and intra-mitochondrial translation [98]. On the same line, Balsa and collaborators [99] used a whole genome gain-of-function CRISPR activation screen in human complex I (CI) mutant cells for the isolation of genes that by gain of function rescue glucose restriction-induced cell death. The study unraveled the cytosolic Malic Enzyme (ME1) as top hit. ME1 was sufficient to allow survival and proliferation of CI deficient cells under nutrient stress conditions. More recently, the same research group applied the “death screen” to search for small molecules able to increase survival of myopathy, encephalopathy, lactic acidosis, and stroke-like episodes (MELAS) cybrid cells. They demonstrated that tetracyclines were effective on mitochondrial disease-derived cells and in a mouse model of Leigh syndrome, which is most often associated with mutations of ETC-OxPHOS genes [100].

#### Identification of HDAC3 and of the Zinc Finger CCCH-type Containing 10 as Mitochondrial Regulators

Our research group also contributed to identifying key regulators relevant in mitochondrial biology, namely HDAC3 and Zinc Finger CCCH-type Containing 10 (ZC3H10). The idea that HDAC3 plays a role in mitochondrial functions stemmed from experiments testing the effect of class I and II HDAC selective inhibitors in obese diabetic mice [101]. In this study, the authors showed that treatment with the class I selective inhibitor MS-275 promotes oxidative metabolism in skeletal muscle, whole-body energy expenditure, uncoupled metabolism in BAT, and favors the transition of WAT to brown fat. In vivo chromatin immunoprecipitation experiments suggested that inhibition of HDAC3 may account for the beneficial effect of the class I–selective HDAC inhibitor. To corroborate the role of HDAC3 on mitochondrial function, a later study demonstrated a peculiar molecular brake action of HDAC3 in maintaining white adipose tissue physiology and functions [34]. It should be noted that HDAC3 has been shown to play a different role in brown adipose tissue, where it is required for the thermogenic program, suggesting a non-canonical coactivator function of this factor [102]. On the other hand, ZC3H10 was isolated as a novel mitochondrial regulator by using a gain of function screen, searching for the ability of 16,000 different cDNA to modulate the mitochondrial transcription factor a (TFAM) promoter activity [103]. 

Between 2010 and 2018, an almost unknown protein, ZC3H10, arose from several studies as an RNA-binding protein (RBP) [104,105]. More specifically, ZC3H10 contains three different CCCH (Cys-Cys-Cys-His) motifs, a glycine rich domain at the N-terminus, and a proline rich motif at the C-terminus [106]. Members of the CCCH zinc finger family have been shown to bind RNA and to be involved in RNA metabolism [107]. ZC3H10 has been described as an RNA binding protein (RBP) that binds mRNA in Hela cells [108], 7-mer synthetic RNAs [109], and microRNA in various cell lines [104]. Due to three zinc finger motifs in the N-terminal region, ZC3H10 can bind not only nucleic acids but also miRNAs [104] and proteins involved in RNA metabolism [110,111]. As reported by Treiber and collaborators [104], ZC3H10 interacts with the primary miRNA, pri-miR-143. Two out of three zinc finger motives are crucial to recognizing the miRNA and, as a consequence, ZC3H10 silencing by CRISPR/Cas9 upregulates 3–4 fold miR143. This mechanism has been observed in several cell lines, including different types of cancer. As regards the biological processes, it has also been reported to behave as a tumor suppressor in MCF-7 cells [112] and as mitochondrial regulator in murine cell lines and human peripheral blood mononuclear cells (PBMCs) [103]. Li and collaborators [113] showed that a basic leucine-zipper domain, positioned between the last zinc finger and the proline-rich domain, was identified in ZC3H10 protein and it was shown to be required for ZC3H10 DNA binding activity. Furthermore, mutational studies demonstrated that ZC3H10 RNA binding activity is not required to activate a thermogenic transcriptional program in brown adipose tissue, indicating that ZC3H10 acts as transcription factor. Moreover, the same group also showed that a disruptor of telemetric silencing 1-like acts as an interacting protein of ZC3H10 and a critical coactivator of thermogenic genes [114]. Recently, our laboratory further contributed to demonstrating the role of ZC3H10 in the transition from mesenchymal stem cells to white mature adipocytes [115]. We showed that ZC3H10 is a pro-adipogenic factor essential to repress protein synthesis and to remodel filamentous actin in the early phases of adipocyte differentiation. This latter mechanism is fundamental to determine a functional population of mitochondria to support the anabolic reactions required during the adipogenic program.

In recent years, different genetic and chemical tools have become available and it is expected that they will enable the identification of novel players of mitochondrial biology relevant to pathophysiology.

## 6. Mitochondrial Diseases and Therapeutic Options: Focus on PGC1α

Mitochondrial diseases are a group of disorders caused by dysfunctional mitochondria. However, dysfunctional mitochondria are also associated with aging and different human diseases such as neurodegenerative and cardiovascular disorders, obesity, diabetes and cancer [1]. The resulting impaired ETC and OxPHOS often leads to a decrease in cellular energy production (ATP), thus, therapeutic interventions aimed at increasing the ATP production available to cells may be beneficial [116].

Although optimal and specific agents are not yet available to improve overall mitochondrial output, there is promising evidence of proof-of-principle and some strategies are in clinical trials. In humans, exercise training, a physiological path to induce mitochondrial biogenesis, improves oxidative capacity in patients with mitochondrial myopathy [117]. In addition, in two different murine models of mitochondrial disease, overexpression of the cristae shaping factor Opa1 corrects the OxPHOS defects and ameliorates the outcome of the diseases [118].

As mentioned above, the control of mitochondrial activity, by the nuclear and the mitochondrial genome, must be tightly and reciprocally controlled to fine-tune key functions in response to extra and/or intracellular signals to adapt to certain energy needs [86,87,88]. The bigenomic control of mitochondria implies that mutations in mitochondrial or nuclear genes lead to mitochondrial dysfunction and consequently to mitochondrial diseases [3,119].

To date, the most detailed prevalence of diseases due to mutations of both the nuclear and mitochondrial genomes has been estimated at around 12.5 cases per 100,000 individuals [120]. Given their complex genetics, mitochondrial diseases can have any pattern of inheritance, including autosomal and X-linked inheritance for nDNA mutations and maternal inheritance for mtDNA mutations. A key difference between nDNA and mtDNA is that mtDNA is a multicopy genome, ranging from just a hundred to a hundred-thousand copies depending on cell type. Some patients with mitochondrial disease are homoplasmic for the mtDNA mutation, however, more frequently patients are heteroplasmic and have a combination of mutated and wild-type mitochondrial genome. In this latter case, the proportion of mutant to wild type mtDNA is important in disease gravity, with higher levels of mutant mtDNA often associated with more severe clinical symptoms. Rare sporadic cases due to de novo mutations have also been reported [3,119].

Mitochondrial diseases belong to a heterogeneous group of genetic disorders characterized by a primary defect in OxPHOS [3,119]. As described above, mitochondrial disorders originate with mutations, acquired or inherited, in mtDNA or in nuclear genes encoding mitochondrial components. The resulting impaired ETC and OxPHOS often lead to decreased cellular energy production (ATP), thus therapeutic interventions aimed primarily at increasing ETC and OxPHOS activities and ultimately ATP levels available to cells may be beneficial [116]. In patients with mitochondrial disease, the clinical symptoms manifestation occurs especially in organs with high energy demands such as skeletal muscle, heart and brain. Symptoms of the disease can arise in isolated organs but much more frequently in several organs at once [119].

In recent years, the genetic diagnosis of mitochondrial diseases has vastly improved, on the other hand effective options for the treatment of these diseases are currently not available. As a result of remarkable progress in three decades of mitochondrial medicine, many potential therapeutic approaches for mitochondrial diseases have been successfully tested in preclinical models [121,122,123,124,125] and others are under investigation in active clinical trials at different stages of development [3,126,127]. Idebenone, a Coenzime Q_10_ analogue used as a tool to improve ETC efficiency, was recently approved by the European Medicines Agency for the treatment of Leber’s hereditary optic neuropathy (LHON) disease (Raxone^®^); however, an international consensus statement suggested this drug for the treatment of patients with acute, subacute or dynamic clinical course, while did not recommend the treatment for chronic patients [128].

Although developing therapies seem promising, rarity, clinical diversity, pathogenic complexity, and the etiological heterogeneity of the diseases, as well as the small number of clinical trials pose several difficulties for the discovery of treatments for mitochondrial diseases [127,129]. On the other hand, our understanding of molecular genetics, pathological mechanisms and clinical manifestation of mitochondrial diseases has significantly advanced, and it still represents the best option to identify innovative targets for the design of ad hoc interventions. Based on the knowledge gained in these studies and on the heterogeneity of mitochondrial diseases, there are still key unmet needs such as guidelines for the treatment of diverse subgroups of patients and the development of more effective and eventually personalized therapies.

This section of the review has the goal to provide an overview of therapeutic options currently in clinical trials for mitochondrial diseases, with a particular focus on those molecules promoting mitochondrial biogenesis, a key process involving PGC1α (summarized in Table 1). Further trials involving other mechanisms such as modulation of oxidative stress, mitophagy, mitochondrial genome, hypoxia, nitric oxide levels and nucleotide pool, are elegantly reviewed by Almannai and colleagues [130].

Physical exercise represents a well-established non-pharmacological option to increase PGC1α levels and mitochondrial biogenesis in healthy and pathological subjects [139,140]. Barth syndrome (Bs) is an X-linked disorder characterized by severe mitochondrial dysfunction, cardiomyopathy, skeletal muscle weakness and exercise intolerance. In this context, a completed clinical trial evaluated the effects of resistance to exercise training on 12 participants (accessible at https://clinicaltrials.gov/by using the National Clinical Trial number (NCT) 01629459, accession on 25 June 2021). Preliminary results from the group responsible of the trial demonstrated that a 12-week endurance (i.e., aerobic) training course increases exercise tolerance only modestly in Bs subjects, with no effect on heart or skeletal muscle function while the training used is beneficial for other populations of subjects not affected by Bs. The blunted effects on Bs may be due to genetic mitochondrial dysfunction in type I (oxidative > glycolytic capacity) muscle fibers. Therefore, the trial focused on targeting type II (glycolytic > oxidative capacity) muscle fibers with exercise training to increase exercise tolerance in Bs participants using a resistance exercise training protocol. The primary outcome of the trial is the evaluation of the changes in exercise tolerance whereas secondary outcomes are focused on change in muscle strength, quality of life, and left ventricular systolic strain. Although the trial has been completed, results are not yet available.

Another approach to induce mitochondrial biogenesis is the use of specific small molecules. Several chemical compounds have been successfully used in pre-clinical models such as agonists of AMPK [141,142], PPARs [121,143], sirtuins [122] and nuclear factor erythroid-derived 2-like 2 (Nfe2l2) [144]. 

The AMPK agonist AICAR (5-aminoimidazole-4-carboxamide ribonucleotide), an adenosine analog, has been successfully tested in mouse models of mitochondrial diseases [141,142]. While clinical trials testing specific effects of AICAR on mitochondrial diseases are not available, two other studies assessing AICAR effects on type 2 diabetes (NCT00168519) and on Lesch–Nyhan disease (NCT00004314), two pathologies characterized by dysfunctional metabolism, have been completed. The primary goal of the trial on type 2 diabetes subjects was the assessment of glucose metabolism, however no results have been so far delivered. Regarding the trial on a single 16-year-old male subject affected by Lesch–Nyhan disease, treated with 30 mg/kg/day of AICAR orally for four days followed by 100 mg/kg/day for four days, along with 400 mg/day allopurinol, no improvements were noted in his neurological, behavioral, or hematological status. These results were probably due to the very low oral bioavailabi1ity (<5%) of AICAR in humans [131]. Another interesting double blind, randomized, placebo-controlled trial attributed to AMPK activation the effect of dark chocolate consumption, an (−)-epicatechin enriched food, on the better exercise performance and increased mitochondrial biogenesis of sedentary subjects. Briefly, 20 normal, sedentary subjects (∼50 years of age) were randomized into placebo or dark chocolate consuming group (20 g/day of dark chocolate for 3 months). Before and after treatment, subjects underwent bicycle ergometry to evaluate VO_2_ max along with skeletal muscle biopsy to assess changes in mitochondrial function, density and oxidative stress. In addition, blood collections were performed to assess metabolic endpoints. Taub and colleagues [132] detected a 17% increase of VO_2_ max in those subjects consuming dark chocolate compared to placebo. Increased levels of HDL and decreased triglycerides were also reported. Of note, significant increases in LKB1, AMPK and their phosphorylated protein forms as well as PGC1α protein levels and citrate synthase activity were also induced in the group consuming dark chocolate. No differences were reported in mitochondrial density. Experiments conducted in vitro or in vivo sustained the role of (−)-epicatechin as a compound capable of inducing mitochondrial biogenesis [145,146,147].

The FDA approved bezafibrate, a pan PPAR agonist, has been used in an open-label, investigator-led, non-randomized experimental trial to evaluate the effects of bezafibrate in patients with mitochondrial disease (NCT02398201) [133]. The primary goal of the trial was to evaluate the ability of bezafibrate to improve cellular energy production in patients with the m.3243A > G MTTL1 (Mitochondrially Encoded tRNA-Leu (UUA/G) 1) mutation. The subjects enrolled in the study received 600–1200 mg bezafibrate daily for 12 weeks. Liver function was normal and nonsignificant side effects were reported. The authors reported a reduction in the number of complex IV-immunodeficient muscle fibers and improved cardiac function. On the other hand, they also observed increased serum levels of fibroblast growth factor 21 (FGF-21), growth and differentiation factor 15 (GDF-15) both proposed as biomarkers of mitochondrial disease [148]. These effects were also accompanied by dysregulated serum levels of amino acids and fatty acids [133]. On the basis of these results, the authors concluded that although bezafibrate has potentially beneficial effects on mitochondrial biogenesis in the short term, the increase in markers of mitochondrial pathology and the altered metabolomic profile raise major concerns about the long-term consequences in the studied population. Another trial, currently terminated due to the COVID-19 pandemic, aimed at testing the ability of a novel PPARδ agonist REN001 in patients with primary mitochondrial myopathy (NCT03862846).

As mentioned above, NAD^+^ (nicotinamide adenine dinucleotide) is a cofactor for SIRT1, which in turn activates PGC1α [48]. Administration of nicotinamide riboside, a vitamin B3 and NAD^+^ precursor, was effective in inducing mitochondrial biogenesis in skeletal muscle and brown adipose tissue in a mouse model of adult-onset mitochondrial myopathy, preventing mitochondrial abnormalities and mitochondrial (mt) DNA deletion formation [149]. *The Role of Nicotinamide Riboside in Mitochondrial Biogenesis* is the official title of an open-label experimental medicine study, currently in the recruitment status, aimed at evaluating the safety, bioavailability and capacity to induce mitochondrial biogenesis of nicotinamide riboside in 15 participants with mitochondrial disease caused by the m.3243A > G mutation in mtDNA. Beside these primary outcomes, the amelioration of mitochondrial disease symptoms will also be assessed as secondary outcome (NCT03432871). Another ongoing randomized, double-blinded, placebo-controlled trial on patients with mitochondrial disease caused by the m.3243A > G mutation in mtDNA and with mitochondrial myopathy is testing the effect of Acipimox, a niacin derivative used as a lipid-lowering agent, on ATP levels in skeletal muscle as a primary outcome. Secondary outcomes include several parameters, among which are improvement of quality of life, VO_2_, VCO_2_, anaerobic threshold, pulmonary ventilation, respiratory exchange ratio (RER), ATP/ADP ratio, NAD^+^/NADH ratio and mtDNA copy number (http://www.isrctn.com/ISRCTN12895613, accession on 25 June 2021). KL1333 has been shown to be a novel NAD^+^ modulator promoting NADH oxidation and capable of activating SIRT1 and PGC1α [150]. A double blind, randomized, placebo controlled, single and multiple oral dose trial, now in the recruiting phase, will test the effect of this novel compound primarily on healthy volunteers to assess safety, tolerability and pharmacokinetic parameters and then on patients with primary mitochondrial disease (NCT03888716). Recently, Pirinen and colleagues [134] showed that adult-onset mitochondrial myopathy patients suffer from systemic NAD^+^ deficiency. The same authors carried out an open label clinical trial by administering increasing dose of niacin to boost NAD^+^ (to 750–1000 mg/day; NCT03973203) in mitochondrial myopathy patients and their matched controls for 10 or 4 months, respectively. The treatment was well tolerated by participants. NAD^+^ levels were dramatically increased in all subjects (up to 8-fold) while pathological patients showed normalization of NAD^+^ to control levels within muscle. All participants showed increased muscle strength and mitochondrial biogenesis. Furthermore, the muscle metabolomic profile of affected subjects was almost normalized to that of control subjects. Finally, niacin treatment also led to decreased whole-body fat percentage in controls and increased muscle mass both in controls and mitochondrial disease patients. The authors concluded that niacin supplementation could be an efficient strategy to counteract NAD^+^ deficiency in mitochondrial myopathy patients.

The natural polyphenol resveratrol was shown to boost OxPHOS and mitochondrial biogenesis trough SIRT 1 and PGC1α activation; it has also been proposed as AMPK activator in vivo and as cAMP-specific phosphodiesterases (PDEs) inhibitor [147,151,152,153,154,155]. Alway and coworkers [135] conducted a randomized controlled trial on 65–80-year-old men and women to test the hypothesis that resveratrol treatment (500 mg/die) combined with exercise would increase mitochondrial density, muscle fatigue resistance, and cardiovascular function more than exercise alone. Resveratrol supplementation coupled with exercise improved mitochondrial density and muscle fatigue resistance more than placebo or exercise treatments alone, thus providing evidence that this strategy might be a better approach for reversing sarcopenia in the elderly. Another double-blind, randomized, placebo-controlled, cross-over study evaluating the effects of resveratrol supplement (1000 mg/day) on physical ability and on muscle metabolism in patients with mitochondrial myopathy and patients with a defect of fatty acid oxidation due to VLCAD and CPTII deficiencies has been completed. However, the results of this trial are not available yet (NCT03728777). Resveratrol and (-)-epicatechin, both natural polyphenols, are thus far effective on mitochondrial function as described above [132,135]. This is of outmost importance considering that Mediterranean diet and especially extra-virgin olive oil consumption represents an extraordinary source of polyphenols. The different polyphenols present in extra virgin olive oil have several beneficial properties, to name a few their anti-inflammatory effects and their ability to counteract oxidative damage. A thorough description of these effects is elegantly detailed in [156,157]. Moreover, a Mediterranean diet and virgin olive oil nutrigenomic approach was previously successfully applied to reduce the expression of proatherogenic genes in human-derived peripheral blood mononuclear cells [158]. Therefore, a similar approach may also be beneficial on mitochondrial diseases and/or on pathologies characterized by mitochondrial dysfunction.

A hallmark of the mitochondrial disease Friedreich’s ataxia is the impairment of antioxidative defense mechanisms, which plays a major role in disease progression. RTA 408 (omaveloxolone) is a potent activator of Nfe2l2 [159], the master regulator of cellular redox homeostasis [160]. In addition, several lines of evidence suggest that Nfe2l2 activation can increase mitochondrial respiration and biogenesis [161]. On this basis, 69 Friedreich ataxia patients were randomized 3:1 to either RTA 408 or placebo administered once daily for 12 weeks at doses ranging 2.5–300 mg/day. The drug was well tolerated, and adverse events were generally mild. This study showed that RTA 408 treatment appears to improve neurological function of Friedreich ataxia patients at the optimal dose level of 160 mg/day, however no data are yet available about mitochondrial biogenesis (NCT02255435) [136]. More recently, results about another randomized double-blind trial with RTA 408 on mitochondrial myopathy patients was published (NCT02255422) [137]. In this study, 53 participants received RTA 408 at 5, 10, 20, 40, 80, or 160 mg, or placebo for 12 weeks. The primary outcome was the assessment of changes in peak cycling exercise workload, while the secondary outcomes were the evaluation of the 6-minute walk test distance and the submaximal exercise heart rate and plasma lactate. RTA 408 at the dose of 160 mg was well-tolerated, however despite the primary outcome results were not successful, the levels of lactate during submaximal exercise and heart rate were reduced by the drug treatment. These data are consistent with improved mitochondrial function and submaximal exercise tolerance [137].

Finally, a subset of mt-tRNAs contain a unique modification derived from taurine, a non-essential β-amino acid found in most animals [162]. Previous studies, nicely reviewed in [163], have demonstrated that taurine modifications are closely associated with mitochondrial disease, including MELAS (myopathy, encephalopathy, lactic acidosis, and stroke-like episodes) and MERRF (myoclonus epilepsy with ragged-red fibers). Furthermore, the addition of taurine to the culture media of MELAS-derived cells was shown to restore mitochondrial protein biosynthesis, improve mitochondrial function and reduce superoxide generation. In addition, oral administration of taurine (0.25 g/kg/day) in two MELAS patients was capable of preventing stroke-like episodes for more than nine years. [164]. Only recently, Fakruddin and colleagues [165] provided the molecular mechanism accounting for taurine modifications occurring on mt-tRNAs. Indeed, they showed that mitochondrial optimization 1 (MTO1) in mammals is an essential protein catalyzing the taurine modifications. Strikingly, lack of MTO1 dramatically impairs mitochondrial translation and activity. Finally, Ohsawa et al. [138] conducted a multicenter, open-label, phase III trial in MELAS patients with recurrent stroke-like episodes treated with high-dose taurine (9 g/day or 12 g/day) for 52 weeks. No side effects of taurine supplementation were reported, and the complete prevention of stroke-like episodes (primary outcome) was reached by 60% of patients. Moreover, taurine also reduced the annual relapse rate of stroke-like episodes and among the 10 patients participating in the study, five showed a significant increase in the taurine modification of mitochondrial tRNA^Leu(UUR)^, the one most affected in MELAS subjects.

To summarize, in this section of the review, we discussed clinical trials aimed at improving mitochondrial biogenesis. This represents one strategy to counteract mitochondrial dysfunction. However, more trials are currently focused on other aspects of mitochondrial biology including modulation of oxidative stress, of mitochondrial autophagy, of mitochondrial genome, restoration of nitric oxide levels and of nucleotide pool and the enzyme replacement therapy for mitochondrial neurogastro-intestinal encephalomyopathy. A current trial for this latter pathology is also testing the allogenic hematopoietic stem cell transplant. Other trials also evaluated the transplantation of healthy mitochondria into patients by enriching with healthy mitochondria derived from donor white blood cells or from placenta the peripheral stem cells of patients, a procedure known as mitochondrial augmentation therapy. All these trials and studies are reviewed in [130]. In the future, we expect that comprehensive metabolomic profiles of plasma and other biological fluids from patients with mitochondrial diseases could aid in the definition of novel, disease-specific biomarkers that can be exploited to monitor the outcome of a given therapy.

## 7. Conclusions

In the previous sections, we summarized current knowledge on the involvement of PGC1 related proteins in metabolic pathways that impact key functions in numerous cells, organs and tissues. Specifically, (i) we summarized the basic biology of PGC1 proteins, (ii) we discussed in some detail mitochondria metabolism and features in different cells and tissues, (iii) we described examples of strategies that have led to the discovery of new mitochondrial regulators and, finally (iv) provided a selection of therapeutic applications based on PGC1 proteins and mitochondrial biogenesis.

Despite the wealth of well-established and newly emerging knowledge in the areas addressed in this review, some issues remain open and need further investigation:

- PGC1α vs. other members of the family: most of the studies published in the field and cited in the present review deal with PG1α. The roles of other members of the family have not been investigated in such detail; moreover, in some cases, biological and metabolic roles have only been inferred based on sequence homology among PGC1 family members. Indeed, in the case of PGC1α and β, most functions are shared by the two proteins, e.g., potentiation of mitochondrial biogenesis and oxidative metabolism. Nevertheless, some differences have been reported, for example the different ability of PGC1α and β to modulate the antioxidant response in enterocytes, with important consequences for cancer susceptibility [166]. Therefore, we expect that a thorough characterization of the functions and mechanisms of regulation of PGC1β, PRC and PERC could help distinguish similarities and differences, with important outcomes in pathophysiology.
- Mitochondria and PGC1 proteins in immune cells: understanding the metabolic phenotypes of immune cells and their functional consequences or relationships is an area of intense research. The main challenge encountered by researchers in the field is the complexity of the system, in terms of variety of cell types involved, multiple stages of development and activation, pathological states and biological contexts in which these cells operate. Enormous advances have been made in the field in recent years, especially in pathological settings such as metabolic diseases and cancer, and more are expected to come. We foresee that the application of cutting-edge approaches, such as cell precursors’ tracing and single cell-based techniques, will allow us to gain a more comprehensive view of the multiple metabolic and functional phenotypes of immune cells. A more precise identification of metabolic features, which translate into functional advantages/disadvantages and pathologic/resolving potential, will help future therapies targeting immune cells.
- Discovery of new mitochondrial regulators beyond PGC1: PGC1α represents a paradigm, either for the strategy that led to its discovery or for the wealth of basic knowledge and practical applications that have stemmed from this discovery. We might be led to think that in future years we will identify no other factor as important as PGC1 in the regulation of mitochondria biology. Nevertheless, mitochondria are still mysterious organelles in some respect, with “secrets” awaiting to be disclosed. In the present review we discussed only a few examples of newly discovered factors with a role in mitochondrial biology, but we expect that others may emerge. Even more importantly than in other fields, in mitochondrial biology the use of metabolomics, fluxomics and metabolism-related assays, along with genomic approaches, other omics techniques and functional assays is required for a successful discovery and complete characterization of new potential regulators.
- Mitochondrial biogenesis-based therapeutic approaches: mitochondrial diseases are a complex group of disorders, characterized by varying phenotypes and symptoms. However, it has become clear that mitochondrial dysfunction is found in many other pathologies, from diabetes to Parkinson’s disease. In the present review, we discussed clinical trials aimed at improving mitochondrial biogenesis, one of the main functions related to PGC1 proteins. Indeed, some trials have led to successful outcomes, at least in part. However, we are now aware that biogenesis is not the only way to fight mitochondrial dysfunction. The so-called “mitochondrial medicine” should rely on the multi-faceted nature of mitochondria and related processes, including de novo mitochondrial biogenesis possibly coupled with mitophagy to assure turnover with more functional organelles. In addition, it should exploit any new knowledge emerging from basic research and implement advanced tools such as enzyme replacement therapy and transplant of healthy mitochondria.

## Figures and Tables

**Table 1:** Studies in humans aimed at promoting mitochondrial biogenesis.

Intervention	Access to the Study	Target Patients	Goal	Status
Exercise	NCT01629459	Barth syndrome.	Target type II muscle fibers with exercise training to increase exercise tolerance.	Completed. No data available yet.
AICAR	NCT00168519	Type 2 diabetes.	Assessment of glucose metabolism.	Completed. No data available yet
AICAR and Allopurinol	NCT00004314	Lesch–Nyhan disease.	Improve neurological, behavioral, or hematological status.	Completed. No improvements were noted in neurological, behavioral, or hematological status. These results were probably due to the very low oral bioavailabi1ity (<5%) of AICAR [131].
Dark chocolate (−)-epicatechin enriched food	Not available	Sedentary Subjects.	Bicycle ergometry to evaluate VO_2_ max and work and skeletal muscle biopsy to assess changes in mitochondrial function, density and oxidative stress. Metabolic endpoints in blood.	Completed. Increase of 17% in VO_2_ max, increased HDL and decreased triglycerides in those subjects consuming dark chocolate compared to placebo. Significant increases in LKB1, AMPK and their phosphorylated protein forms as well as PGC1α protein levels and citrate synthase activity in the group consuming dark chocolate. No differences were reported in mitochondrial density [132].
Bezafibrate	NCT02398201	Patients with the m.3243A > G Mitochondrially Encoded TRNA-Leu (UUA/G) 1 (MTTL1) mutation.	Improve cellular energy production in mitochondrial disease.	Completed. Liver function was normal and nonsignificant side effects were reported. Reduction in the number of complex IV-immunodeficient muscle fibers and improved cardiac function. Increased serum levels of fibroblast growth factor 21 (FGF-21), growth and differentiation factor 15 (GDF-15) both proposed as biomarkers of mitochondrial disease. These effects were also accompanied by dysregulated serum levels of amino acids and fatty acids [133].
REN001	NCT03862846	Primary Mitochondrial Myopathy	Assessment of REN001 safety in subjects with primary mitochondrial myopathy.	Terminated due to COVID-19 pandemic. No data available.
Nicotinamide Riboside	NCT03432871	Patients with m.3243A > G mutation in mtDNA.	Evaluation of the safety, bioavailability and capacity to induce mitochondrial biogenesis	Recruiting. No data available yet.
Acipimox	ISRCTN 12895613	Patients with m.3243A > G mutation in mtDNA.	Evaluation of ATP levels in skeletal muscle and several other parameters among which improvement of quality of life, VO_2_, VCO_2_, anaerobic threshold, pulmonary ventilation, respiratory exchange ratio (RER), ATP/ADP ratio, NAD^+^/NADH ratio and mtDNA copy number.	Ongoing. Expected conclusion of the trial early 2022.
KL1333	NCT03888716	Healthy volunteers and patients with primary mitochondrial disease.	Assessment of safety, tolerability and pharmacokinetic parameters on healthy volunteers and then evaluation of mitochondrial parameters in patients.	Recruiting. No data available yet.
Niacin	NCT03973203	Patients with mitochondrial myopathy.	Capability of niacin to activate dysfunctional mitochondria and to rescue signs of mitochondrial myopathy.	Completed. NAD^+^ levels were increased in all the subjects and pathological patients showed normalization to control of NAD^+^ levels within muscle. All the participants showed increased muscle strength and mitochondrial biogenesis. Furthermore, muscle metabolomic profile of affected subjects was almost normalized to that of controls. Niacin treatment also led to decreased whole-body fat percentage in controls and increased muscle mass both in controls and mitochondrial disease patients [134].
Resveratrol	Not available	Men and women 65–80 years of age.	Ability of resveratrol treatment combined with exercise to increase mitochondrial density, muscle fatigue resistance, and cardiovascular function more than exercise alone.	Completed. Resveratrol supplementation coupled with exercise improved mitochondrial density and muscle fatigue resistance more than placebo and exercise treatments [135].
Resveratrol	NCT03728777	Patients with mitochondrial myopathy and patients with a fatty acid oxidation defect of VLCAD and CPTII deficiencies.	Investigate the potential beneficial effects of a daily supplement of resveratrol on physical ability and on muscle metabolism.	Completed. No data available yet.
RTA 408 (omaveloxolone)	NCT02255435	Friedreich ataxia.	Ability of RTA 408 to activate Nfe2l2 and modified Friedreich’s ataxia rating scale (FARS) and to change peak workload during exercise testing.	Ongoing. RTA 408 treatment appears to improve neurological function of Friedreich ataxia patients at the optimal dose level of 160 mg/day. No data about mitochondrial biogenesis available yet [136].
RTA 408 (omaveloxolone)	NCT02255422	Patients with mitochondrial myopathy.	Assessment of changes in peak cycling exercise workload and evaluation of the 6-minute walk test distance and the submaximal exercise heart rate and plasma lactate levels.	Completed. RTA 408 at the dose of 160 mg was well-tolerated. No changes in peak cycling exercise workload. Lactate levels during submaximal exercise and heart rate were reduced by the drug treatment. Data are consistent with improved mitochondrial function and submaximal exercise tolerance [137].
Taurine	Not available.	Patients with myopathy, encephalopathy, lactic acidosis, and stroke-like episodes (MELAS).	Capability of taurine supplementation to prevent stroke-like episodes.	Completed. Complete prevention of stroke-like episodes was reached by 60% of patients. Taurine also reduced the annual relapse rate of stroke-like episodes and 50% of patients showed a significant increase in the taurine modification of mitochondrial tRNA^Leu(UUR)^, the one most affected in MELAS subjects [138].

## Data Availability

Not applicable.
